# The Administration of Medical Relief to the Poor, under the Poor-Law Amendment Act, &c.

**Published:** 1842-04-01

**Authors:** 


THE ADMINISTRATION OF MEDICAL RELIEF TO THE POOR, UNDER THE POOR LAW AMENDMENT ACT, AND OTHER LEGISLATIVE PROVISIONS FOR THE PUBLIC HEALTH, CONSIDERED IN THE REPORTS OF THE POOR-LAW COMMITTEE OF THE PROVINCIAL MEDICAL AND SURGICAL ASSOCIATION. To which are Appended certain Clauses suggested for Insertion in the Contemplated Bill for the further Amendment of the Poor-Laws. London, Sherwood and Co. 1842.

UNFORTUNATELY, too many of *our* readers are acquainted with the manner in which the administration of medical relief to the poor has been conducted. The Poor Law Amendment Act, whatever may have been its merits of principle, has, in many important particulars, been infamously mismanaged in detail. Unnecessary interference, repulsive want of feeling, and gratuitous cruelty, have characterised its operation. Inhumanity wears its severest front, when sanctified by abstract principles. It then becomes a species of fanaticism, and dries up the wells of human kindness, in bosoms, where at other times they may freely flow. Whoever doubts this, need only look at the proceedings of the Commissioners, and Assistant Commissioners, under the New Poor Law. The brutality of the old parish overseer might have been more revolting from its coarseness, but was infinitely less mean, equivocating, and contemptible than that which we have lately seen in high salaried officers and reputed gentlemen. A pertinacious adhesion to arbitrary rules in spite of all remonstrance, and in defiance of experience, has not been more conspicuous on the part of the Commissioners, than their shuffling out of the consequences of their acts. Their harshness has only been exceeded by their meanness.

Nor has their policy the merit of success. They came into office with the evils of the old poor law fresh in men's memories. The new law which they had the task of administering had many admirable features, and was calculated both to regenerate the poor, and to benefit the rich. If it called up some sinister interests against it, that is what every social change has to encounter, and such an opposition cannot long resist the force of public approbation. Some years have elapsed and how do the Commissioners and the new law stand now? The only thing which can exceed the unpopularity of the one is the odiousness of the other. No public functionaries in England are so much disliked. Their defence has overthrown one government, and will, unless concessions are made, impair the stability of another. By the poor they are detested—by the rate-payers they are either positively disliked or coldly tolerated—by the medical profession they are regarded as in the light of natural enemies—and by the press they are denounced in the most opprobrious terms. If their position had difficulties, the Commissioners have not been the men to diminish them.

Their treatment of the medical profession has been contumelious and unwise in the extreme. We foretold, some years ago, that the Commissioners in attacking the medical profession, were raising up numerous and powerful enemies against themselves and the law. The event has shewn that we were right, and has demonstrated the evil as well as the folly of unnecessarily multiplying opponents to the measure. But that is the affair of the Commissioners, who think perhaps that they are so marvellously loved that nothing can shake the good opinions of their countrymen.

We propose to take a retrospect of some of the doings of these Commissioners, and to exhibit their consideration for the poor, as well as their courtesy towards the profession. We shall probably place them in a pleasing light, for which we can take no merit to ourselves, their own actions speaking for them too decisively to require much in the way of comment.

1. The first point to which we shall allude is the size of the Unions. On this the Commissioners plumed themselves, no doubt, seeing how difficult it has been to reduce them. The admirable consequences of assigning the smallest possible number of doctors to the greatest possible number of patients, spread over the greatest possible space, might be anticipated à priori. No doubt the Commissioners and their creatures act on the same principle in their own cases, and don't care to have the medical attendant of their families within some eight or ten miles of them. That being the fact, the following was only Christian conduct towards their fellow-paupers. We quote one instance out of many.

"The AYLESBURY UNION was described as being 'principally situated in the vale of Aylesbury, which consists of a damp, clayey, retentive soil, subject to inundations in wet seasons;' the atmosphere 'cold and humid;' the occupation of the working men chiefly agriculture, that of the women lace-making. The wages being low, 'their circumstances are bad, their physical condition is inferior, and their intellectual endowments small; they are a poor sickly race of beings, which is partly attributable to the soil, and partly to their insufficient diet.'

The principal acute diseases prevalent among the poorer classes in this Union, are 'intermittent, remittent, and continued fevers; affections of the mucous membranes, often epidemic to a great extent.' 'The chronic diseases are scrofula, nervous affections, indigestion and other diseases of the stomach,' which are remarkably prevalent. These peculiar features of the Union were detailed to shew the necessity for a careful provision of relief to the sick poor.

The Union was formed in July, 1835, by Mr. Assistant-commissioner Gilbert. Its shape is particularly irregular and inconvenient. It contains a population of 21,480, and forty parishes, which the guardians distributed into four districts.

Sixteen medical men, who previously attended these parishes, signified to the board of guardians, through one of their number as deputy, their readiness to continue their services, provided their salaries could be equitably adjusted, one in each district becoming responsible for three or four others who would assist him. The guardians, however, instructed by the commissioner, and following a practice but too frequently before adopted in the locality, had already *advertised for tenders,* which were to specify a payment per case, diminishing in amount as the total number of cases increased, with a maximum limit to the salary.

The resident practitioners stated their objections to this unjust proposal, which, professing to remunerate the medical officer according to the duty performed, deprived him of the miserable pittance when called upon for an unusual amount of exertion. They stated that they should have less objection to a payment per case, if the obvious principles of such a mode of remuneration were not violated by the limitation of a maximum; they therefore proposed fixed salaries for the four districts, amounting to £595; or, in case this were refused, they were prepared to accept a sum equal in amount to the average cost of medical attendance for the last three years, in the several parishes comprised in the Union.

The guardians rejected this most reasonable offer, insisting on a rigid adherence to the conditions of their novel and absurd proposition.

The medical men, averse to such a scheme, made one more attempt at conciliation, by suggesting a minimum limitation of the salary, as a protection or set-off against the maximum which the guardians required for their own security from the demands of disease and destitution.

But the board rejected all compromise, and the districts were disposed of, the first to a candidate residing out of the Union (not one of the sixteen), and the other three to two adventurers brought from London with tenders, in conformity to the prescribed conditions. The terms on which these districts were taken varied from 2s 3d. to 4s. per case, according to the number of patients, limited by a maximum of £525.

Rather, therefore, than pay the somewhat higher salary first proposed by the medical men, or incur the average medical expenses of the previous three years, until a better system could be substituted,—or even rather than meet the subsequent endeavours of the profession to some approximation of terms, the Board of Guardians wantonly sacrificed to their own ignorance and pertinacity, the legitimate interests of a number of established practitioners; not content with this, they withdrew the poor from the care of their tried medical advisers, and handed them over to persons of whose character, principles, and talents, they knew nothing.

The subsequent history of the medical contracts in this Union is instructive.

District No. 1, the greatest diameter of which was sixteen miles, was at first intrusted to a medical officer who also held a district in another Union (Tring), in which he resided. The most distant point from him was eight miles, at which point medical advice could be obtained within four miles. In the second year, another surgeon, residing out of the district (at Aylesbury), was appointed, from whom the most distant point was twelve miles! In the *third* year, *a third* medical man held it, together with another district (No. 2). This person was one of the London strangers, and resided at the same place with the preceding surgeon.

In this unfortunate district was exhibited not only the evil of repeated annual changes, but a progressive deterioration, for three years, in the medical arrangements.

The other districts suffered nearly similar mutations.

The greatest diameter of district No. 2 was fourteen miles. It was held by the same medical officer, a stranger from London, who, during the first two years, also held district No. 4, and in the third year district No. 1; this enormous extent of duty being undertaken without any assistant! The remotest part of the district was eight miles from his residence; but he might have been called upon after accomplishing fourteen miles of this district to travel nineteen miles through No. 4.

District No. 3, containing fourteen parishes, and nine miles in diameter, was taken for the first year by the other London adventurer, who settled in a central village. The second year, another surgeon just commencing practice in the same place succeeded him in office. In the third year, the guardians appointed a fresh practitioner residing out of the district, his residence being distant from its two extremities not less than seven and eight miles.

District No. 4 was held during two years by the medical officer of No. 2, the *nearest* point to whose place of abode was seven miles, the remotest twelve. Medical assistance might have been obtained at four points within a moderate distance of *all* the parishes. In the third year this district was held *singly* by *another surgeon* recently introduced, and residing at the same preposterous distance.

According to the understanding with the committee, the consequences of these arrangements were not detailed in evidence, it was only stated that 'cases of fever and acute diseases occurred which could not be visited properly; and elsewhere accidents occurred which could not be properly attended to.'

But a reference to the minute book of the board of guardians would disclose lamentable reasons for the dismissal of some of their oft-changed medical officers, and for the official reprimands which even the most determined indifference to the sufferings of the sick poor could not repress.

During the first year two inquests were held on patients who died under the treatment of the new surgeons; but the exculpatory verdicts by no means quieted popular suspicion.

No means were neglected to maintain the credit and the position of the strangers, and, in several instances, patients, under the gratuitous care of the established surgeons, were compelled to relinquish their aid, and apply to the Union medical officer in order to become entitled to any pecuniary relief.

Your Committee possess the details of neglected cases in remote parishes, where the paupers suffering from acute and serious disease were literally unable to procure medical aid." 10.

In the Eton Union, one of the districts, containing a population of

5585, and six parishes, formerly attended by four medical men, each having a competent assistant, and each living near his charge, was entrusted to *one* medical officer, a stranger, who was located at the distance of six, seven, and even eight miles from the remote parishes.

The FAVERSHAM UNION was formed in 1835 by Sir Francis Head. It contained a population of 14,845 souls, and twenty-five parishes, and was forty miles in circumference.

Eight medical men, all competent and qualified, had previously attended these parishes, at an average expense of about £500. per annum. On the formation of the Union, the assistant-commissioner advised the board of guardians to offer £250, without assigning any reason for so extraordinary a reduction. The resident practitioners, desirous of being employed, declined this offer, and proposed the average annual amount of the former salaries. The board of guardians would listen to no compromise, and advertised the medical care of the Union at the above sum, £250.

A perfect stranger, being at the time in prison for debt, applied for and obtained the appointment to the whole Union! He was without a horse or an assistant, and destitute of the necessary remedies and means of preparing them. In consequence of his inferior equipments, the board reduced his salary to £225, thus incapacitating him still further for attending properly to his unfortunate patients.

The complaints of the poor were loud and constant. Nevertheless the guardians retained their medical officer until 1837, when, from increasing embarrassments, and inability to fulfil his engagement, he abruptly absconded.

The HAMBLEDON UNION, formed in March 1836, by Mr. Mott, contains 11,882 inhabitants and sixteen parishes, which were previously attended by seven practitioners, residing in and dispersed over the Union, the extent of which is twenty miles by ten, and the area about 106 square miles. The average annual salaries had been about £350, and the extra charges nearly £100. more.

After the usual "palaver" with the medical men, about as dignified and satisfactory as one on the banks of the Tchadda, advertisements were issued for "tenders," when one was sent in at £110. for the whole Union, by a person from a distance, who had passed his examination only a year and a half previously. The guardians added £40. to the sum specified in his tender, and actually appointed him to the care of the entire Union! The following year they added £100. to his salary, making in all £250, and soon after allowed him extra remuneration for midwifery, the very terms proposed by the former surgeons.

We may well suppose that this fortunate gentleman can do justice to his *poor* patients spread through the moderate area of 106 square miles! He must be, like the ghost in Hamlet, hic et ubique.

The SHIPSTON-UPON-STOUR UNION contained a population of 19,030, and thirty-seven parishes, in which *ten* medical men at least had formerly the charge of the poor, at an average annual cost of about £500, including extras.

There was the average (perhaps rather more) amount of shuffling, terminating in the appointment of one medical attendant to the whole Union. Mr. Assistant-Commissioner Stevens distinguished himself upon this occasion. Of course the poor suffered, but that was no great matter. The deuce of it was that "them wicious paupers," as Mr. Bumble very justly denominates them, would not die in quiet, so, in the third year, the guardians found it necessary to deprive their protégé, of one of the four districts; and, in the fourth year, the complaints reached such a height, that the Union was re-divided, and four medical men appointed, whose collective salaries amounted to £310.—an arrangement which still subsists.

In another part of the pamphlet before us, we find some further evidence of the convenient size of districts deduced by Mr. Farr from the Parliamentary returns. That gentleman states in evidence:—"The Newbury Union is seventy-two square miles in area, the Leighton Buzzard Union is fifty-five square miles. The surgeon appears to reside nearly in the centre of the district, but it is eight miles from his residence in one direction. District 2 of the Oakhapton Union, fifty-four square miles, and the boundary eight miles from the surgeon's residence. In the Northleach Union, the area of two medical districts is 109 square miles; the surgeon resides out of the district: apparently it is eleven miles from his residence to the southern extremity. In Northumberland, the Haltwistle Union, two districts, comprise 108 square miles. In Westmorland, Shapwest-ward comprises ninety-eight square miles; the distance from the surgeon's residence in one direction is nine miles. In York, the Driffield Union comprises 115 square miles, its population 14,718."

Mr. Farr also mentioned the immense population of several other districts. For example, the Dover medical district comprised a population of 20,507; the Sevenoaks and Shoreham district a population of 13,735; Leicester Union, district No. 1, 23,954 inhabitants; Bethnal Green Union, divided into three districts, comprised a population of 62,018. Other districts were alluded to, which, though not excessive in population and area, presented serious obstacles to a prompt supply of medical relief. Thus, "in the Ledbury Union, district No. 2, the surgeon appears to reside out of the district. The distance from his residence is eleven miles in one direction, and ten miles in another. So in the Leominster Union, district No. 3, the surgeon resides out of the district, the distance of the boundary from his residence being 12 miles in one direction. In the Hereford Union, district No. 3, the distance is ten miles. In the Broughton Union, Lancashire, it is eleven miles; in the Calton district, twelve miles; in the Clun Union, eleven miles." Several similar instances could be given.

Whatever commissioners or assistant-commissioners may say, we fancy that there will be but one opinion on this matter. And yet the commissioners defend such preposterously cruel arrangements. They reported that—"No sooner was any part of the country formed into a union, than the propriety of altering the arrangements established for medical relief in the separate parishes, and of resorting to a new and more combined distribution, became apparent." This thing which is so apparent they do not, however, prove, but stammer out a sort of shuffling apology. Thus, Mr. Gulson alleged "that the medical men under the old system rode over the ground which other medical men had in their districts." This assertion may be met at once by the well-known fact, that, on the introduction of the new system, the riding over each other's ground was vastly increased, by separating the private and pauper practice, and employing two surgeons in a parish, where one had previously sufficed. Then, the commissioners, taking shelter behind the guardians said, " the districts have been deliberately formed by the respective boards of guardians, who, from their local knowledge, must be considered to be the most competent judges on the subject; and we have reason to believe, that in almost every instance the best arrangement was adopted." Now, to controvert this specious statement, made, be it observed, in 1836, when the abuses were at their height, it is only necessary to refer to the parliamentary evidence, especially to the early distribution of the parishes in the Aylesbury, Lincoln, and Shipston Unions, in all of which the guardians shewed the folly of their arrangements by abandoning them. They did not, however, abandon them merely from a sense of justice. That would ill befit either guardians or commissioners. It was only when a few coroner's inquests had occurred, and made things awkward and unpleasant. Then indeed these provident guardians, on whom the commissioners, good easy souls, depended, thought it prudent to give in, the rates having been lightened, in the interim, of two or three sickly paupers.

All these excuses are a mere fetch. So far from a new arrangement being necessary, no evidence was adduced to shew that any one parish was either destitute of, or at an unnecessary distance from, medical advice; on the contrary, cogent reasons were brought forward in support of a system, which made it the interest, personal and pecuniary, of the rate-payers of each small country parish to place their poor under the care of that practitioner whose residence was most convenient, and whose practice brought him, most frequently, into the village. The inhabitants of rural districts did not require an assistant-commissioner, unacquainted with their locality, to direct them whither to send for medical advice.

The real object of the commissioners and of their creatures had no relation to the benefit of the poor or the convenience of the surgeon. They advertised large districts, because they were calculated to *stimulate competition*. They seemed worth having, that was the real motive. To put the sick poor up to auction with effect, they must be put up in droves. And certainly they went in goodly lots. Poor serfs, too many of them were literally *knocked down* to the lowest bidder.

Mr. Gulson, the considerate gentleman who advocated large districts because in little ones the medical men rode over one another's ground, finding, perhaps, the faculty not quite so grateful as it ought to be, threw in *his* plea for the poor. And a very kind one it is. He professed that he could not understand why *the poor*, in cases of extreme illness, should suffer more from the distance of medical advice than *other* sick persons. The testimony of the medical witnesses would have informed him. " In the first place, they have to send to the relieving officer and to the surgeon, a distance of seven, eight, ten, eleven, or fourteen miles; and after they have sent for the surgeon they have to send for medicine, or the person has to wait at the surgeon's house until he returns and orders the medicines......... If the distance were less considerable, younger children might be sent; but when the distance is so great, of course a grown up person must be necessarily employed; and that causes a great waste of the labourer's time." Nor is the injury confined to the poor. The increase of expense to the surgeon is immense: the difference between visiting patients scattered over a small and a large district must be very great: it would involve the keeping of two or three horses, in addition to the horses which he would require for his private practice, It has a direct tendency to increase the expense of the medical attendance, whether that expense is borne by the surgeon or the guardians is indifferent; it is an unnecessary expenditure of time and labour which might be otherwise employed.

If Mr. Gulson's wits were so dull it is a pity that he ever accepted the situation of an assistant-commissioner. But we doubt if Mr. Gulson is quite such a noodle as he would have us believe. We dare say it occurred to him or his superiors that poor people a long way off from the doctor will be very likely to neglect their ailments, rather than be at the trouble of fetching him—while the doctor will be not unlikely to neglect them too, rather than be at the trouble of going. So two very desirable things are obtained—medical relief is curtailed without the inconvenience of refusing it, and a few of the most obstinately invalid paupers, bid, like Richard's patients, the world good night.

To sum up, the formation of these monster districts never operated, and never could be intended to operate, as a boon to either the poor or the profession. They were formed in order to coerce the one, at the expense of the health and lives of the other. A very humane and sensible proceeding.

One other point we cannot refrain from noticing. At the very time, and in the very Unions where the frightful consequences of the system were exhibited, the commissioners obtained from the guardians, who had been their accomplices or tools, evidence of its admirable working. This was duly published in their annual report, and we do not doubt that it is quite as trustworthy as the remainder of their documents. In the North Aylesford Union the guardians reported the medical arrangements as "adequate to the relief of the paupers." In the Wallingford Union they pronounced them as "quite adequate, and equally efficient with the former arrangements." In the Eton Union, "adequate and decidedly better." In the Faversham Union, "quite as adequate and more efficient." In the Hambledon Union, as "adequate and as efficient." In the Tendring, "adequate." In the Woodbridge, "quite adequate, and quite efficient." In the Aylesbury Union, "adequate and efficient." And (will it be credited?) the guardians of this Union, who were at length compelled to dismiss two of their medical officers for neglect or improper conduct, had the assurance to report, that only two or three complaints were made, which, on investigation, proved to be without foundation!! In the Bridgewater Union, the disgraceful system adopted was described by its authors as being "adequate and more efficient." Adequate no doubt it was, and more efficient, too, for never before were there such inquests in England for mal-practice.

The next point to which we shall advert is the palpable insufficiency of the remuneration under the new system.

Mr. Farr calculated, from the returns made to the parliamentary committee, that "the amount of remuneration in the metropolitan unions was on the average 1*s*. 5¼*d*. per case; in Lincolnshire, 5*s*. 4*d*. per case; in

Dorsetshire, 3*s*. 6*d*.; in Devonshire, 3*s*. 5*d*.; in Cheshire and Lancashire, 6*s*. 1½*d*.; in Wilts, 1*s*. 11½*d*.; and, taking the average of these counties, 3*s*. 3½*d*. per case."

He first proved how totally inadequate this was, by a calculation of the prime cost of medicines, which cannot be properly supplied to pauper patients under 2*s*. 6*d*. per case.

In the metropolitan districts, therefore, the sum paid to the medical officers was 1*s*. per case less than would suffice to supply the patients with drugs alone; whilst in the country districts, on the average, only 9*d*. was allowed for the time and labour of the medical attendant, and the cost of journeys. In some counties, indeed, the remuneration was absolutely below the proper cost of medicines.

That 2*s*. 6*d*. per case is barely sufficient to provide drugs for the poor was the opinion of other witnesses. Mr. Ceely said, "I should be sorry to furnish all the medicines for the Aylesbury Union at 2*s*. 6*d*. per case." Mr. Farr stated, on the authority of Dr. Bigsby's pamphlet, that "the cost of medicines in twenty-two dispensaries was 2*s*. 1½*d*. per case. The Apothecaries' Company supplied the navy with *galenicals* at 2*s*. 3½*d*. per head," equivalent to about 3*s*. 6*d*. per case; the addition of *chemicals* would, probably raise it to about 4*s*. per case. It was shewn that the cost, in many hospitals and infirmaries was much higher; for example, in St. George's Hospital it averaged 5*s*. 2½*d*. per case, for all the patients, in the years 1834 and 1835, but the "average of nine county hospitals, including both *in* and *out* patients, was 3*s*. 7*d* per case," and, according to the Rev. C. Oxendon's "Statistical Report of Provincial Hospitals," the average cost of each patient in drugs, leeches, wine, spirits, and surgical instruments, was 3*s*. 11¼*d*.; the expense in drugs and leeches only, 2*s*. 5½*d*. per case.

The Committee of the Association are quite disposed to agree with Mr. Farr, that "as the result of a careful examination of all the returns given in—medicines, leeches, bottles, bandages, and medical appliances of every kind, would amount to 5*s*. per case, on cases of all descriptions, such as they occur in private practice, of perhaps fifteen or twenty days' duration; and that, with the greatest economy, applying Mr. Chadwick's dietetic principles to the Materia Medica, the cost of drugs for medical cases of twenty-three days' duration, cannot be fixed at a lower sum than 2*s*. 6*d*.; the patients could not be supplied at 2*s*. 6*d*. with the same medicines precisely as are supplied to the rich; but I can conceive, that by various expedients, without withholding essential drugs from the sick poor, the cost of medicines may be reduced to 2*s*. 6*d*."

It is quite evident that if the remuneration does not cover the actual cost of the medicines, the poor will not have justice done them. It is not to be supposed, human nature has not arrived at that ideal of perfection, that medical men, or any other men, will incur loss permanently and continuously for charity's sake. But granting that they might do it voluntarily, it is certain that they would not do it on compulsion. Well-fed, well-salaried officials exercise a power which the law has inadvertently placed in their hands, and force a profession into a position of indignity and pecuniary loss. Is it likely that, smarting under a sense of injury, they will discharge the duties of their position well? Common sense replies that they will not. The lives then of numbers of our fellow-creatures are made the subjects of a flagitious bargain and inhuman parsimony.

Did the commissioners set us a worthy example—did they renounce emolument and profit—did they endeavour to reduce their own bloated salaries to a minimum, we might then submit to privations with more cheerfulness, and cease to murmur at economy, which was, at all events, impartial. But we look in vain for a self-denying ordinance on the part of any of these gentlemen: their care for the pockets of the rate-payers begins below themselves, and the screw is applied just at that point where their own knuckles are safe.

Yet the ingenuity so profitably occupied in taking care of themselves, has not been altogether withheld from us. These gentlemen would have it believed, that low payment is a capital thing, and that a man is making most when he is money out of pocket. Thus, the chairman of the committee (he got the hint no doubt from the commissioners) was desirous of extorting admissions, that as the effect of inefficient treatment would be to protract or aggravate diseases, it must be the interest as well as the duty of the medical attendant to provide properly for the cure of any patient. But the witnesses proved that a medical officer, if "determined to get through his duties at the least possible expense and trouble, has the means of so doing by administering cheap and inefficient remedies, without its being discovered either by the patients, the guardians, or the public;.... that it is in the power of the practitioner to neglect his duties to a very great extent, perhaps to produce a prolongation of suffering, and very frequently loss of life, without his delinquencies being detected by unprofessional observers;" and, therefore, that a person of this description might, for a time, fancy it to be his *interest* to deprive the poor of the necessary remedies.

Certainly it was a happy idea and worthy of the poor law commissioners to stimulate a medical man to do his duty by paying him so badly that every case was a loss. It is stolen from the Chinese plan of stopping the physician's salary while the patient is sick. But it is an improvement upon that—for the Chinese, if they mulct the doctor during illness, pay him fairly in health. The poor law commissioners recompense us neither in one nor in the other.

Pray do these gentlemen adopt this plan in the cases of themselves and their own families? Does "good advice" mean the lowest or the highest priced? Does a man expect worse or better attendance for a guinea fee or a half-crown one? If the above precious reasoning were worth anything, the duration of the case ought to be in the ratio of the amount of remuneration. A man who should consult Sir Benjamin Brodie for his knee, should pay him shillings instead of sovereigns. This would make Sir Benjamin so sick of the case that he would cure him off hand on purpose to get rid of him.

The obvious fallacy in this mode of reasoning is the assumption that attention is cheaper than neglect. Anybody who knows anything of physic is aware that it is not. It would *cost* the medical man much more to cure a child by leeches, than to let it die by negligence—to cure a case of cachexia with sarsaparilla, than to let it go on in sæcula sæculorum with bread pills. The poor law commissioners first engage needy men by the pressure of competition, and then render the contract so ruinous that it is impossible they should perform it. And the sufferers, be it recollected, are not merely the improvident or dishonest parties who contract, but the guiltless and the helpless poor.

The felon is better off than the pauper. Mr. Farr shewed that the average cost of medical attendance upon prisoners in the English jails was 13s. 7d. per case, and in the Irish jails still higher. Hence it might be inferred, that the State values the life and health of a felon at four times that of a pauper, since the remuneration of prison surgeons bears that proportion to the payment of union surgeons. If the readiness of the latter to accept office on inadequate terms be the cause, as Mr. Gulson intimates, of so extraordinary a discrepancy, the salaries for prisons might, with equal propriety, be reduced by professional competition to the same level. And why have they not? Simply because the ill effects of such a course had been previously discovered. "The medical attendance on prisons was formerly conducted very much with a view to cheapness; but when an enactment was made, which shewed that it was the wish of the legislature to have efficient services, and pay for them adequately, the visiting magistrates raised the terms of remuneration to a fair extent, and have by this means pretty generally now rendered the appointment of surgeons to prisons one that the most respectable members of the profession feel desirous of obtaining." This reform was described by Mr. Farr in the following terms:—

"The consequence of neglecting the prisoners in jails has been made too frequently evident to allow any parties to overlook the fact. Jail fevers and other diseases have often attracted the attention of government, and the number of deaths occurring in the jails are recorded and known to every body; deaths occurring over a scattered district, and at isolated points, are often forgotten, and make no general impression; and again, on the other hand, the magistrates appoint the surgeon to the jails, and the magistrates are not so difficult to deal with as the old overseers were, and they are rather more scrupulous."

"The overseers (and he might have added *the guardians*) attended more to the amount of the salary, than to the efficiency with which the duty was performed."

Mr. Power, the assistant-commissioner, candidly confessed that "he did not see any reason why, with reference to those parties who are provided with medical attendance by the public, any distinction should exist as to the expense between those who are provided for by the government and those by the parish."

The humanity of public bodies depends, we fancy, on the pressure from without. It is a sort of barometer, falling when public opinion is stagnant and dull, rising when it becomes clear and expressive. Felons get good medical advice, because if cut off by jail fevers, great folks who are in contact with them are frightened, or coroner's juries and the press are troublesome. But the village pauper dies unheeded, and the tenant of the cabin may rot without the aid of public cognizance or sympathy. So that what one might call the head-money of the cottier comes to be four times less than that of the pickpocket or burglar.

The following additional statements will put in another, though hardly in a stronger light, the injustice of these arrangements.

"The standard of union medical salaries has hitherto been estimated with reference to the number of cases attended, but the real cost per case depends of course upon the duration of the attacks of illness. In different localities, and in different types of disease, no less than under different systems of bestowing relief, it is evident that the average duration of cases must vary greatly. The calculation per case is therefore not always an accurate index of the rate of remuneration. Perhaps a more satisfactory method of demonstrating the inadequacy of parochial payment was that suggested by Mr. Farr, by ascertaining the cost of *one* patient sick for a year. It appears from his evidence, that for a pauper constantly sick, or for a succession of single cases, the average cost was £2. 13s. per annum, according to the returns from the eight counties. Now, by the army regulations, country surgeons, in the absence of a military surgeon, are paid at the rate of 4s. 4d. per annum per man for the medical care of the troops, except where the number is under fifty, when the rate is increased to 6s. per head. Taking the number constantly sick at 4 per cent., the payment for each sick soldier per annum would be either £5. 8s. 4d. or £7. 10s. according to the numbers; the lowest of these rates being more than double the amount of pauper medical remuneration, without taking into account the expense and loss of time caused by journeys to distant parishes.

The disparity of payment observed in various unions was very singular, and, as Mr. Farr stated, could not be explained by anything in the returns. In Devonshire the payment per case ranged from 1s. 6d. to 8s. and 10s. apparently without reference to area or distance. Thus, also, in the Wycombe Union, Bucks, the remuneration per case was 2s. and 2s. 9d.; whilst in the Bridge Union, in Kent, it was 10s. per case per quarter, so that, in cases lasting a year, it would amount to £2.

Neither commissioners nor guardians appear to have proceeded on any fixed principle in determining the payment per case; but in order to guard against the consequences of too liberal an estimate, they almost uniformly limited the number of cases to be paid for; thus they secured themselves by a maximum of the cost, whilst they left the medical officer with a certain prospect of loss, either by their effecting a reduction in the number of his cases below the proposed maximum, or by granting orders to a greater number of sick than they meant to pay him for.

The gross unfairness of such an arrangement was shewn by some of the medical witnesses, but the point is too obvious to require further notice in this Report.

The remuneration under the new system was asserted by Mr. Gulson to be relatively greater than before, owing to the reduction of medical pauperism. He supposed this to be the case, 'because only one-half of the people that used to be attended are so now; the remainder provide themselves with medical attendance.'

Mr. Gulson was never more mistaken. An unprejudiced inquiry would have convinced him, that gratuitous aid supplied the deficiency of parochial charity, and that the medical paupers continued so still, though they were dignified by the name of independent labourers, because the parish no longer bore their expenses.

Even those who professed a wish to pay for medical advice found themselves unable to do so, and the losses sustained by attendance on the labouring classes were much increased." 36.

In fine, the new system ground down those who took the unions—imposed a tax on the humanity of those who would not take them—and left the sick poor to half-paid incompetence, or unpaid spontaneous, and therefore uncertain benevolence. It wronged the pauper, the profession, and its own protégés. The latter indeed have been much in the plight of those who sell their souls to the devil. The devil always manages to get the best of the bargain.

We now come to the TENDER SYSTEM, and a tender system it is.

The poor law commissioners assert in their second annual report:—"We have *never sought to disturb or displace the medical practitioners in their respective districts.* Whenever this has been done, (and the introduction of individuals from a distance has occurred in but a very few instances), the guardians have been forced to adopt that course by the inadmissible demands which have been made upon them by the gentlemen who now complain of their practice having been interfered with."

The Report before us quietly and conclusively establishes the contrary. The demands of the medical practitioners were based on the old scale of remuneration on which the commission on the old poor laws remarked, that "medical attendance seems in general to be adequately supplied, and economically, if we consider only the price and amount of attendance." That could not be considered inadmissible, the reasonableness of which had been thus admitted. But the commissioners proceed in the same jesuitical strain. They repeated before the parliamentary committee that "the instances in which strangers have been introduced, bear a small proportion to the whole number of the unions;" and so, according to Mr. Gulson, "the introduction of a stranger was quite the exception." The obvious reply is, that its being the exception, instead of the rule, originated not in any favourable disposition of the authorities to the established practitioners, as events have sufficiently proved, but in the fears and necessities of the latter.

The fact is, that, in the vast majority of unions, some of the resident medical men sent in "tenders," or submitted to the terms dictated by the guardians, not because they considered appointment by tender defensible, nor because the offers of the guardians were less inadequate than in those unions where strangers were introduced, but because they were resolved, at all hazards, to keep out another rival; or occasionally, it is feared, were desirous of occupying a position which would enable them to infringe on the private practice of their established competitors. The guardians were, indeed, seldom reduced to the necessity of introducing persons from a distance. The mere threat of such a proceeding frequently availed to ensure acquiescence on the part of the resident practitioners. Examples of this are to be found in the Banbury, Chipping-Norton, Cookham, Eastry, and Reading Unions.

Sir Francis Head, the assistant-commissioner, openly boasted that the immense reduction, which he had made in the medical salaries, was principally effected by the free use of such threats. It can scarcely be believed that the commissioners have persuaded themselves, although they endeavour to persuade others, that the apparent acquiescence of the medical men in so many unions, *thus obtained*, was any proof of their cordial approval of the arrangements of the guardians.

Nor should such statements as the following pass unnoticed. "Many medical men, who set their faces against these arrangements in the first instance, now cordially accede to them." A reference to the unions beforementioned will prove that, although, in many instances, the established practitioners, tired of the contest, and smarting under the injuries inflicted upon them, have yielded to the guardians, and accepted office on almost any conditions; yet in nearly an equal number, such has been the change in the opinions and proceedings of the guardians, that the medical gentlemen have been enabled to hold the appointment with a due regard to their own consistency. In these cases the guardians have amended their system, not the medical men their views.

All this is of a piece with the conduct of the commissioners throughout. First they cajole, then, if that does not answer, bully the medical practitioners. Many this intimidates—some it stings to resistance. Those who are intimidated are held up as evidence of the satisfaction which the system gives. Those who resist are said to be unreasonable in their claims—then to have no ground for their complaints because their claims have been conceded. Whilst the matter is confined to country parishes, the commissioners brag and bluster—when the public and the parliament become arbiters, the actors change their dresses, and then all is subterfuge and equivocation. A more sneaking exhibition of tyranny and humbug we have never witnessed. But to proceed.

"The advertisements for 'tenders,' the dictation of terms below the previous inadequate remuneration, and the imposition of degrading and injurious conditions, such, for instance, as the establishment of a medical club, were direct attacks on both the legitimate interests and the character of the profession. One of these, at any rate, was necessarily sacrificed, and the miserable alternative forced upon every parish surgeon, either of degrading his professional character by furnishing tenders, and accepting discreditable terms, or of forfeiting his fairly-earned advantages, by surrendering the care of the poor to strangers. Under these circumstances, therefore, what ground for surprise or censure is afforded by the fact, that the medical residents collectively have acted on the defensive in many unions, and occasionally, no doubt, with some asperity?" 21.

The thing to be regretted both on the profession and the poor's account, is not the existence of combination, but the want of it. It was not wide enough. Had the profession been true to itself, the commissioners must have been defeated. It is very well for those commissioners, armed with authority, and using it in the most offensive and arbitrary manner, to declaim against that combination which alone offered any chance of resistance. The outcry is another specimen of the *humbug* of which we have seen so much. Combination is neither a good thing nor a bad thing in itself—it is the object which that combination is to serve which stamps it as criminal or praiseworthy. What are some of our very best institutions but combinations or unions? What is the whole machinery of commissioners and assistant-commissioners and boards of guardians but a combination of the rate-payers? And shall only the powerful combine, and the weak not be suffered to unite? If the medical practitioners attempted to enforce unreasonable claims and improper demands, then their combination was criminal. But if those claims were just, and those demands moderate, and such they have been proved to be, the combination in support of them was laudable and necessary. The misfortune is that it was not more extensive and more successful.

We quite agree with the Report before us that the proper course for the boards of guardians to pursue at first, would have been to invite the medical men practising within their respective unions (such at least as were properly qualified) to meet and assist them in the medical arrangements, offering them the aggregate of the former parochial expenses, and acting on their suggestions in appointing the medical officers, distributing the parishes, and apportioning the remuneration. The attempt to effect all this on Mr. Gulson's plan, without the cordial co-operation of at least the majority of the professional corps, could only terminate, as it has done, unsuccessfully. Had an opposite course been pursued, the contest might have been avoided; the heart-burnings, the severance of ancient connexions, and the social animosities, which have resulted from the desperate measures adopted by the poor-law authorities, would not have been heard of. Time would have been afforded for preparing the data on which to found an improved system of medical relief, and the guardians would not have been obliged, as now, to retrace their steps, and practically acknowledge their error. That the course here indicated would have been pursued in many unions, is clear, had not the poor-law commissioners prevented it. They ought rather to have enforced it in all.

Nor can it be said that the medical practitioners were wedded to the old system. They acknowledged its abuses, which may be easily summed up:—the absence of medical superintendence, and, indeed, of responsibility to any proper authority; the frequent adoption of tender in the appointment of parish surgeons; the degrading nature of the competition thus called into action among medical practitioners; the want of adequate inducement to perform properly the important duties of the office; lax and insufficient attendance on the poor; the operation of the settlement laws producing and encouraging charges for attendance on non-parishioners, which charges, if not *absolutely* high (of which there is no proof), were so *relatively* to the pitiful salaries awarded by overseers and select vestries; the occasional employment of ignorant and unqualified practitioners; the absence of any sufficient check on the appointment of distant surgeons, who from motives of speculation might offer their services; the indefinite nature of the liabilities of the medical officer, producing a tendency on the part both of the rate-payers and of the poor, to extend these liabilities at his expense, and to the injury of all parties concerned,

But the poor-law commissioners seem to have regarded the profession and the paupers in nearly the same light. They put the screw upon both. The policy as well as justice of their measures are already pretty apparent. An unpopular body of functionaries at the best, they have wantonly damaged by their precipitancy, self-sufficiency, harshness to the poor, and disregard of the feelings of the public, the success of the measure entrusted to them. So that after some years' trial it is more hated than ever, and the prolonged existence of the commission itself is in very imminent peril.

The crowning insult as well as wrong inflicted on the profession was the mode of carrying out the "tender system." The commissioners, when they first adopted this, thought they had the profession in their power. They were aware that our ranks are crowded, that many are found in them oppressed by poverty and forced by necessity to disregard the suggestions of that nice sense of honour which only flourishes in affluence. They believed that they need only raise the standard of competition, and mercenaries would flock to it. To a certain extent they were successful. But of the adventurers they enlisted, some deserted a service which they found both dishonourable and ruinous—others displayed their own incompetency and the gross impropriety of the whole arrangement—while the voice of the entire profession, backed by the indignation of those who were shocked at the working of the system, gradually acquired a degree of force that made itself heard in parliament and out of it. The commissioners put upon their trial resorted to the old game of subterfuge and equivocation, and cast about for any excuse that might serve to hoodwink the public.

Thus, Mr. Gulson in his evidence, and the commissioners in their reports and other documents, endeavoured to shew that, at the commencement of their operations, the guardians had no alternative but to require tenders. They said, "it is only by resorting to open tender, that, situated as the guardians are, in the formation of new unions, they can ascertain, with any thing like correctness, the sum which it may be right to pay for the medical relief of a district."

Mr. Gulson stated: "Tender has been unavoidable, and is a good plan in the first instance to ascertain what we can fairly get it done for;" and, "In highly pauperized districts, I decidedly recommended tender, because we found it difficult to fix a sum;" and again, "We could not avoid it in the first instance," qualifying his assertion, however, by excepting "those Unions in which the sum that had hitherto been given appeared to be moderate." Mr. G. thus admitted that it was unnecessary to resort to tenders in every instance, consequently the commissioners stand corrected by the statements of their assistants. In fact, the commissioners did fix salaries in Kent and Essex, and the departure from the "tender" system was about as just and as conciliatory as the adoption of it. They fixed a sum according to Mr. Gulson, where pauperism prevailed and the medical salaries were consequently "high." The Reporters observe that this appears to have been the mode of reasoning:—"where the paupers are few, a calculation may be made, because they are not worth putting up to auction; but where they are numerous, a fair calculation would raise the salaries, which it is my aim to depress: competition must, therefore, be encouraged; highly pauperized districts are probably the result of the 'high' medical salaries; the number of paupers must, therefore, be reduced by diminishing the remuneration of their attendants. Sickness and destitution will decrease where relief is withheld."

The shallowness of this excuse was too palpable and it was dropped. Then another was shuffled up.

"'The elements,' wrote the commissioners in their second Report, 'on which the calculation must be founded are in themselves utterly unknown to the persons who are selected for the office of guardians; the medical practitioners themselves cannot fail to be possessed individually of the knowledge necessary for making the calculation, and in asking them to bring it forward in the way of tender, nothing more was meant than that they themselves should in the first instance suggest the amount of the reward, which, in their own view, their services might entitle them to,—thus, in truth, constituting the medical practitioners, and not the boards of guardians, the judges of the fitting amount of remuneration for their attendance.' In like manner the chairman of the committee suggested that 'the guardians ascertained by tender what in the opinion of the medical men, being themselves competent judges upon this subject, their remuneration should be.' There are two assumptions in this specious but shallow apology for tender: first, that the medical men who furnished them possessed the *data*, as well as the 'knowledge necessary for making the calculation;' secondly, that the only method of inducing the medical practitioners to furnish such a calculation was to require tenders.

The fallacy of these assumptions was shewn to the parliamentary committee. The great majority of practitioners were at that time without sufficient information as to the elements of the calculation. The subject had *then* been investigated by but few. The average cost per case of a proper supply of drugs had not been correctly estimated. The writings of Dr. Calvert, Mr. Smith of Southam, and our late lamented colleague, Mr. Yeatman, had given very inadequate notions of the prime cost of medical attendance and medicines for the poor; and even these imperfect calculations were unknown to the bulk of the profession. So defective and objectionable was the old system, that it disposed even medical men to undervalue parochial services, and therefore to underrate the expense of proper medical relief.

The former surgeons of parishes knew, indeed, that they had never been remunerated. 'Their contracts had never been founded on previous fair calculation of the number likely to be relieved, and the real cost of careful attendance;' consequently they could never have reckoned on a direct compensation for their time, outlay, and personal exertions.

The introduction of the new arrangements threw additional obstructions in the way of any attempts at such calculations. They knew not for what proportion of the population the guardians might choose to provide medical relief. They could only refer to their old stipends, and take into consideration the increased trouble, which, under the new system, they would incur from onerous returns and inconvenient districts.

But, in truth, whatever the ignorance of the guardians, or of individual practitioners, on the subject, the commissioners had access to some very important sources of information, partly communications made to themselves, and partly valuable papers in the Appendix to the Report of the previous Commission of Inquiry. Had they honestly adopted the valuable and disinterested suggestions contained in these documents, and availed themselves of the assistance of the medical gentlemen in the several unions, they might have framed plans far less objectionable, without any recourse to tenders." 27.

What the commissioners knew they kept to themselves, or used that knowledge with the unworthy view of trepanning the profession. To the profession they made their jesuitical appeal. Affecting to ask them to name their terms, they, in reality, imposed their own. To every fair and ordinary bargain there are two parties, and mutual agreement. But the proceedings of the commissioners bear no such just and reasonable aspect. In the language of the report, "Were the medical practitioners addressed as parties without whose cordial co-operation *no* system, whether provisional or permanent, could be properly administered? Were they supplied with such data for the calculation, as the commissioners and guardians alone possessed? Were respectable and long resident practitioners consulted? No:—instead of such a wise and magnanimous course, the commissioners chose to appeal to every individual in the profession, whether resident or non-resident, employed or unemployed, established or seeking a home and a morsel of bread. All were invited to transmit their estimates, and the prospect of office was held out to that person whose estimate of the fitting amount should most nearly coincide with the views, and appear best calculated to promote the designs, of the commissioners. Let any unprejudiced person pronounce whether a project could have been devised more likely to destroy every notion of adequate remuneration, in the minds both of those requiring, and of those furnishing, tenders.

The poor-law commissioners were fully aware that the sums specified in these tenders would not bear any relation to the value of the article supplied.

How much less discreditable, then, would it have been, had they openly avowed, that their sole object in requiring tenders was to reduce the union expenditure, and not descended to evasions so palpable as to strike even the most simple !"

The defence of the commissioners is, as usual, tricky and fraudulent. "The guardians have, in very many instances, been induced to set the lowest tender aside, solely with reference to considerations of character and personal qualifications." "I have seen numberless instances where the lowest tender has not been accepted." So *said* Mr. Gulson, but his memory was too short or too convenient to cite any instances of such magnanimity. We should like indeed to see them. Generosity and the poor-law functionaries are so wide apart, that one is apt to think they could never come together. We would wager something that could any seeming instance of such liberality be brought forward, there would be found some shabbiness at the bottom of it—some paltry specimen of favouritism or spleen.

But if there have been no instances adduced of the rejection of the lowest tender—there have been enough of its acceptance. In the Wallingford Union, for example, a district and a single parish were, *by the direction of the assistant-commissioner, Mr. Stevens,* entrusted to two medical men living at a distance, who had sent in the lowest tenders, instead of to the resident and tried practitioners, whom the guardians wished to re-appoint. Yet, in the face of such facts, the commissioners ventured to assert that the boards were not required to accept the lowest tender. And in the Crickhowell Union, a young man, who had resided in Wales only four years, and understood not a word of the language, having sent in the lowest tender, was appointed to the care of one half of the Union, "where you may go for miles before you would meet with a person who could interpret between the medical man and his patient." In fact, the examples are too numerous to cite, and too notorious to require it. And the object was served, for the knowledge that the lowest tender might and in all probability would be taken, rendered every tender ruinously low. The "tender" system was the bullying system, and as such the commissioners employed it.

But soft. These *gentlemen* never in their innocence conceived that the system was unpopular. In their second annual report, they allege that it was never supposed such a course (viz. requiring tenders) was derogatory to the character of the profession. The simple souls. We dare say they thought that the profession *liked* being put on a par with the contractors for the parish bread and beef. There was something quite inspiring in that. And medical men were very fond, no doubt, of being bullied into pecuniary loss. The commissioners would have us more quiet even than

Balaam's ass. He *did* speak when he was smitten. The reporters justly observe—"So mistaken and degrading an estimate of the character of the medical profession shews the total unfitness of the commissioners to undertake the management and regulation of its concerns. They must have been well aware that such a practice would not have been tolerated by either of the other learned professions, nor in appointments to any other public offices. Their conduct towards medical practitioners was, therefore, without excuse."

We only trust that, as the tender system appears so unobjectionable to the commissioners, their own offices will be filled by it. We are quite sure it would work well there. Any change *must* be for the better.

But even to this, or to any other system, except cajolery and intimidation, which appeared in some shape at every place and under every disguise, the commissioners were "constant never." The reporters observe, that, without acting upon any well-defined or acknowledged principle, the commissioners excepted certain unions from the operation of pecuniary competition, "constituting" either themselves or the guardians "judges of the fitting amount of remuneration,"—that, in other unions, where the tenders which they had invited exceeded their expectations, they compelled a reduction of the amount, by threatening to introduce strangers, —and that occasionally they persuaded the most eligible candidate to accept office at the salary specified in the lowest tender.

And what reason and gentlemanly feeling and justice would alike combine to reprobate, what practice too has condemned, a parliamentary committee, biassed in favour of these arrogant commissioners, has explicitly and irrevocably damned.

> "If offers have been made and appointments accepted by the resident surgeons at a rate below a reasonable amount of remuneration, under an apprehension that strangers to the neighbourhood might be introduced, and that a part of their private practice would be lost, together with their attendance on the poor, your committee think that this is a circumstance to be regretted, and they advise the adoption of some different mode of appointment." 30.

So the "Tender System" has got its death-blow, although its spawn are left behind.

We believe that it is unnecessary to say more on the subject of the inadequate remuneration of the medical men, improper size of their districts, and injustice of the tender system. But there are other grievances behind, not less real because less apparent.

1. One of the principal evils of the old system of parochial medical relief was the want of efficient supervision and control of the medical practitioners employed to attend the poor.

On the introduction of the new poor-law, the commissioners endeavoured to supply this defect, by requiring weekly returns of the diseases affecting the paupers, of the attendance of the medical officers, and even periodical reports of the treatment of cases. But the reporters observe that this plan proved both ineffective and offensive: *ineffective,* because neither the commissioners nor the guardians were competent to decide whether a sufficient amount of medical attendance had been afforded in any given case; and *offensive,* inasmuch as "highly qualified practitioners could not feel satisfied in submitting their practice to the judgment of non-professional persons;" whilst the weekly attendances at the board, required of the medical officers, were felt to be derogatory, unnecessary, and incompatible with regular professional engagements.

The art, and, we may add, the impotence, of the commissioners, are palpable. They first cram, nolens volens, an obnoxious office on the practitioner. Sensible that forced into it by necessity its duties must be irksome, and the losses it entails, if possible evaded, the unhappy officer is treated with suspicion, and fettered by directions equally useless and annoying. How, in Heaven's name, can a board of guardians be judges of the proper treatment of cases—how can they answer that the real treatment is recorded? They may reply, that to practise such deception would redound little to the honour of the reporter. That is true, but we must take human nature as we find it, and the surest way to debauch men's principles is to insult their weakness. Those who are oppressed by force learn, too soon, to resist by fraud.

A general report is proper enough, but to erect a quorum of village dogberries, or even the luminaries of Somerset-house into arbiters of medical or surgical treatment is about as offensive as ridiculous. Their attempting to arrogate this as well as all other authority is a downright piece of impudence.

But the commissioners, perhaps, have nearly caught a tartar.

"The recommendations of the medical officers with regard to the diet of the sick, although generally complied with, soon began to excite alarm in the minds of economical guardians. The gentlemen, who imagined themselves competent to decide on the amount of attendance required in illness, would not feel at a loss in determining the propriety of 'full diet.' Occasionally, however, they were in a dilemma, as appears from a case suggested by the chairman of the committee. There might be two districts in a union, 'the climate, circumstances, state of health, &c. being the same,' in which the medical officers 'might differ widely in their dietetic directions.' One might be a follower of Brown, the other of Broussais, and the guardians, in the exercise of their authority, would feel it incumbent on them to pronounce judgment between the parties. The decision would of course prove any thing but satisfactory, especially to the Brunonian. Dr. Kay, in reply, advised that the guardians should avoid any direct interference with individual cases, but merely summon the medical delinquent before them, and give him a 'general admonition.' The case of a refractory medical officer was next supposed, and here Dr. Kay could suggest no remedy but dispensing with his services 'at the end of his annual engagement.' Then, again, the chairman of the committee was anxious to know what should be done if there were no other resident medical practitioner competent to undertake the care of the union, or disposed to obey the wishes of the guardians relative to the diet of the sick. Dr. Kay could see no other alternative than to import a medical man from London or some distant district, 'though the necessity for such a step would be regarded as a misfortune.'

This part of the evidence suggests several important queries; for instance—Should the medical officer be compelled, under pain of dismissal, to adapt his mode of practice to the opinions of his unprofessional employers? Should his inadequate remuneration expose him to the suspicion of ordering meat and beer, instead of expensive tonics and stimulants? On the other hand, should the guardians be compelled to furnish the precise amount of diet which any medical officer might see fit to prescribe? Should the latter, in fact, be constituted the *absolute* dispenser of parochial relief to the sick?" 51.

Here the shoe pinches. If the medical officer is not paid the cost of drugs, is he to do that by physic at his own expense, which may be done by beef and beer at the expense of the parish. We protest that he is a fool if does so. If the parish screw him, let him screw the parish. That is the lex talionis, the law of nature. Then see how, in an economical point of view, the matter stands. The medical officer, being badly paid, doles out insufficient medicine and attendance. The case is prolonged. The weakness that supervenes on acute or waits on chronic disease is aggravated, and then the parish pays in bread and beef and beer. That this has been the history of many a case we do not entertain a doubt, nor do we hesitate to prophesy that if the present scale of medical remuneration is persisted in, the rate-payers will be losers rather than gainers by it. Where there's a will there's a way, and if the medical practitioners are treated as they have been, we are much mistaken if a mode is not discovered of taking revenge.

Dr. Kay's threat is that of an old woman. What does it signify where his pet doctor is procured? The system is bad, and protégé of the commissioners or not he feels it so. Is it likely that an adventurer without money and without principle would be slow at expedients to reimburse himself. Why that is the very man who would be most inclined and indeed compelled to resort to them. No Dr. Kay. The way to ensure moderation and fairness is to practise them. And depend upon it that if you do not, the natural order of justice that seems established in the world will in some irregular fashion vindicate itself. If the rate-payers remunerate the medical officer he will act considerately by them. But if they wrong him, they may depend on it they will have no great reason to chuckle.

One word to our medical brethren. We advise them not to curry favour with the guardians. Let them report manfully every abuse. If they do not they will get the devil's pay and the devil's thanks, and have the pleasure of finding themselves the first victims. Let them remember the Seven Oaks Union. If, then, there is over-crowding, insufficient clothing, imperfect or improper nutriment, proclaim it. Let there be no squeamishness in advising good rations and stimulants if necessary. If the union won't pay for physic let it pay by all means for food.

The tenure of office, by annual contract, has been a source of complaint. It was looked upon as tending to diminish the value of union appointments in the estimation of respectable practitioners, and to discourage a zealous performance of duty. It was likewise shewn to be irreconcilable with the principle so generally admitted, that a public officer should continue to hold his appointment "*quamdiù se bene gesserit.*" Even Dr. Kay, in apparent forgetfulness of these considerations, suggested that workhouse appointments, "being deemed desirable by the profession," might be held *in rotation* by several medical gentlemen during the year. But there appeared to the committee no sound reasons for depriving the poor of a medical attendant who had obtained their confidence, and had properly fulfilled his duties.

Without pretending to limit the duration of the appointment to one year, we rather think that the term should not be a lengthened one, supposing that the office were made, as it should be, a desirable and honourable one. To give it to one person, however meritorious, to the exclusion of his competitors would smack of monopoly and partiality. We think that all should enjoy the appointment in their turn, although we conceive that that turn should not come round too frequently.

The commissioners in this, as in every thing else, asserted their right to display the most despotic caprice. In April 1836, Mr. Secretary Chadwick, in reply to an inquiry of Mr. Wetherhead (a candidate for a medical office in the Strand Union) stated, "by order of the board," that the appointments of paid officers are *not* annual, but during good behaviour, &c. The same official personage, in April 1837, informed Dr. Webster, also "by order of the board," that the appointments of medical officers are annual. We suppose that these bashaws do not think it necessary to explain or justify these opposite sic volo sic jubeo decrees.

The next point to which we shall advert is the formation of medical clubs. The crooked policy of the commissioners is well set forth in the report before us.

"Their main object appears to have been gradually to restrict the administration of medical relief within the same limits as relief in money or kind. In order to effect this, they found it necessary to induce or compel that numerous body of semi-paupers, which had been long accustomed to the former species of relief, 'to provide it for themselves.'

Besides the many *indirect* means adopted to attain this end, such as districts of large size, medical officers previously unknown in the locality, and inadequate remuneration, already noticed, there were some of a *direct* kind, the first of which, and the simplest, was the restriction of medical relief to a smaller number, by refusing 'orders.' Mr. Gulson admitted that, in the pauperised districts, 'not one-half of the people were attended that used to be.'

In many unions the disposition to deny relief was favoured by the adoption of a payment per case; a mode of remuneration which made it the interest of the parish authorities, and the duty of the relieving officers, not to grant orders for medical attendance, if they could avoid it.

As may be supposed, the consequences of such a system were frequently serious, and even fatal. They were only partially and occasionally mitigated by the inclination of the poor to seek gratuitous aid, and the humane readiness of the profession to bestow it.

Many of the sick poor were therefore left to the natural progress of disease. Yet Mr. Gulson did not hesitate to assert, that the authorities exercised their discretion with regard to medical relief 'humanely and very liberally.' How is this to be reconciled with his admission just quoted? Even were it expedient to refuse *one-half* of the former recipients that assistance to which they had been accustomed, most certainly it was neither 'liberal' nor 'humane.'" 54.

The dirty scheme of the commissioners is palpable. First they insult and injure, in every way and by all their arrangements, the medical profession—then they endeavour to deprive the pauper so far as they can of the ill-paid and often inefficient advice they have procured for him—and, lastly, they throw on the charity of the profession those to whom their own is denied. Anything more paltry than this cannot well be conceived on the part of public functionaries. And what right has the state or the state's salaried servants to presume in this fashion on the charity of any class of men? Our charity (it is notoriously great) is our free will gift—it is not the creature of compulsion, nor is it meet that it should be. If we choose to exhibit it, good—the feelings that it gratifies are its reward.

But even charity must not flow at the bidding of those who make a market of it, and justice, above even charity itself, commands us in such a case to hold our hands.

But we do the system wrong. In some unions orders for medical relief were given in less niggard fashion. Yes, they were distributed with liberality wherever the contract was at a fixed sum, and where any increase in the number of orders was not followed by a proportionate addition to salary.

It was remarked by one of the medical witnesses, that " where a fixed salary is adopted, the orders are given so freely that a proper discretion is not used with regard to the circumstances of the parties who receive them."

The next method of providing medical relief for the poor, at the expense of the medical men, was the establishment of medical clubs. The commissioners being bent, by hook or by crook, to reduce the price, and we may fairly add, the quality of medical relief to the poor, found it difficult to wheedle the profession into so suicidal an arrangement. They soon, however, devised the means of overcoming this obstacle. By making the establishment of a club one of the conditions of a medical contract; by determining the rate of remuneration in that club; and by threatening to appoint a stranger in the event of objections, they succeeded, in some places, for a time in carrying their point. It appears, say the reporters, that the guardians hoped to secure a twofold advantage from the establishment of a medical club: first, by admitting a class of poor subscribers, they might get rid of a number of applicants for medical relief; and secondly, by annexing the independent club to the pauper contract, they might increase the importance of the appointment, and reduce the terms of the contract.

Such was the view with which medical clubs were established. These poor-law commissioners and their creatures would almost seem to have conceived a malignant spite towards the profession, and to have racked their ingenuity to affront and injure it on every side. First they cut down the remuneration (it is farcical to use the term) for medical attendance upon paupers. Secondly, that not being wrong enough, they clapped on it the additional one of endeavouring to compel the class above the pauper to pay for medical attendance in the same coin. Now observe how this was effected. Mr. Power states in evidence :—" In the parish of Kirtling, the doctor's salary, including every thing, was formerly £15. per annum. The union now only pays him £5. i. e. 2s. per head for fifty individuals on the permanent sick list; but there are eighty families, subscribers to the independent club, which, at 4s. per family, adds £16. to the medical officer's stipend: *he* therefore gains £6. by the alteration, and *the parish* £10. There are other parishes where the plan succeeds equally well for all parties; and I observe that in those parishes *where the medical officers are ill paid,* either no pains have been taken by the guardians and parish authorities to form independent sick clubs, or the poor have themselves formed them, and appointed the doctors to whom they had hitherto been accustomed, instead of the medical officers of the districts, who may happen to be most popular. *This latter practice is certainly some drawback to the means of remunerating the medical officers of the union.* The boards of guardians can, and of course do, recommend *their own* officers to the independent clubs; but beyond that, it would be very impolitic to interfere."

The guardians thus endeavoured to apply the contributions of the "independent" poor to the payment of the union medical officer, who was required, by his contract, to furnish medical relief to the contributors. Yet did the commissioners describe it, as an effort of the poor "to provide, *out of their own resources,* good medical attendance in case of sickness."

These "resources" were evidently *not* "their own;"—"the actual means of obtaining medical relief do not come from the poor," when a certain system, to which they are virtually compelled to contribute, is forced upon their medical attendants.

The small sum, paid periodically by the "independent" members, was not the price of medical advice, but *a sort of composition,* which exempted them from the serious delay and annoyance of seeking an order from the relieving or parish officer, to which the other paupers were subjected Their real dependence on the guardians and on the medical profession continued as before; "they were obviously still *paupers,* as every one must be who is dependent for assistance on the expressed or implied condition of a parish contract."

Again, then, a dirty scheme—professing one thing, effecting another—charity on its lips—tyranny in its operation—zeal for the independence of the poor its avowed, economy at the profession's expense its real object. First, the labourers are coaxed or bullied into forming (save the mark!) an *independent* club—then the medical men are told that if they do not officer the club, that is, be out of pocket for it, there shall be a "tender," and "a talented young man from London,"—then if the unhappy doctor is frightened into attending the club, his union pittance is reduced to its minimum or kept there. If this is not, in a small way, tyranny, we do not know what is. For look at the remuneration to be got from these clubs. In a circular, the commissioners proposed as the minimum contribution, 3s. per annum for a single person; 4s. for a man and his wife; 1s. 6d. for the first child, and 9d. each for the other children. The aged and infirm parents, and the idiots and cripples of every age, were to be paid for as children. Mr. Power, likewise, recommended the same scale, with this difference, that each child was only to incur a payment of 6d. per annum, and every person in the same family, above the age of sixteen, 2s. per annum. The average contribution per head in large families would thus vary from 1s. 2d. to 1s. 6d. The infirm and the helpless were insured at a lower premium than the able-bodied and healthy. The greater the liability to sickness, the smaller was the contribution. A man with a large and sickly family subscribed per head less than half, or one third, of the sum paid by the healthy labourer, who was thus made to contribute indirectly to his neighbour's relief.

If this is not pauperism of the shabbiest sort, what is it? It is cheating the pauper into the belief that he is not a pauper, a thing possible with some thick-skulled chawbacon, and trying to cheat the doctor into the belief that he is paid. But the flam is too palpable for the latter to be gulled, and he is forced to close with a bad bargain secretly resolved to evade it. The poor then, again, are the sufferers; and again they have to thank the penny wise and pound foolish system which presses on them. The reporters remark :—The principle of the clubs is that of "a contract between the poor and a medical person, without any one to protect them. The amount of duty performed, and the cost of medicines furnished, are never made known; the medical officer is the irresponsible receiver of the payments of the poor. The results of this plan therefore, with regard to the treatment of disease and the rate of medical remuneration, are concealed. If the surgeon be humane and honest, he would probably consider it derogatory to his professional standing to acknowledge the ridiculously small sum received for his visits and medicines; if his character be of an opposite description, he would of course refuse to divulge the injustice and neglect which in many instances his patients had endured."

The object of the clubs, however originating, is to depress the scale of medical remuneration—the club itself is a combination against medical interests. It is very well for the fashionable physician or consulting surgeon in extensive practice to smile at these annoyances of their less fortunate brethren. But what is sport to some is death to *them*. Derived from the less affluent classes their income is seriously threatened indeed by combinations and clubs. Look, for instance, at the rate of medical remuneration in some of the most flourishing self-supporting dispensaries. The average cost per case for medicine and medical appliances, surgeons' and dispensers' salaries, was in the Coventry Dispensary (six years) 4s. 5d.; in the Burton on Trent, (two years) 4s. 5½d.; in the Derby, (six years) 3s. 4½d. It is scarcely necessary to remark, that these sums form no approach to a remuneration, even in dense populations. How, then, can they be recommended in rural districts, where area and distance have to be considered?

In Leeds, not long since, the society of "Odd Fellows," 13,000 strong, advertised for medical officers from London, because their former surgeons would not consent to attend them at 2s. 6d. per head per annum.

We hear a great deal of the atrocity of the combinations of workmen, and of their dictation to the masters. But this virtuous indignation all evaporates and disappears when such combinations and such dictation are addressed to the medical profession. Its services are not entreated, but demanded with circumstances of injury and offence in the hour of sickness and distress.

The natural consequences have followed. On the one hand discreditable competition—on the other, unpleasant though necessary combination. By appointing only one surgeon to attend a club, competition of a most discreditable kind has been called into action, and, no less than under the "tender" system, the honour of the profession has been unscrupulously sacrificed to the interest of the individual. In the same locality might be seen several surgeons, having their separate clubs, contending who should demand the lowest rate of subscription, and disgracefully canvassing for members. And thus institutions, professedly designed for the benefit of the poor, became instruments of degradation to the profession. But bad as combination may be, it is preferable to competition, and to combination the profession must be driven, if such serious inroads on its interests are threatened by poor-law unions and poor-law clubs. And they are fools or worse, traitors to themselves and to their brethren, who desert a combination so defensive and so necessary. Depend on it that in combination, pursued with steadiness, and based on nothing but what is equitable, lies the sole security of all. As the reporters observe, the establishment of a medical relief society should be the act of a *united* profession;—of the medical corps in each locality, not of individual practitioners.

We pass over one branch of this entangled subject, or, at all events, intricate report, that, we mean, which contains the suggestions offered to the parliamentary committee by the medical witnesses. But we cannot conclude without quoting the closing remarks of that committee, in *their* report to the House of Commons.

"Your committee," said they, "from a feeling of respect for the medical profession, and believing that their attendance on the poor has been marked by great liberality and humanity, are anxious that the suggestions which have been made by them should be favourably considered by those who are charged with the administration of the law. They recommend the evidence which they have received on this subject to the attention of the poor-law commissioners; and they cannot but hope that arrangements may be made to remove some of the objections reasonably entertained to the present practice, and to put this branch of relief on a footing which shall be satisfactory to the medical men, and be conducive to the comfort of the poor." 80.

Many of our readers may have had occasion some time in their lives to have waited on a man of distinguished rank. They must have felt the difference between the looks and the address of the great man's porter and the great mans' self. The former, all consequence, impertinence, and strut, seems to say "what the devil do *you* want here?" The latter displays the affability of a gentleman, and exhibits the surest indication of his rank by his disinclination to assume it. The contrast between the house of commons and the poor-law commissioners is just as marked and just as ridiculous. The members of the house of commons are gentlemen and act as such. The commissioners and the greater number of their understrappers have exhibited all the arrogance of a parish beadle, without the set off of his portly person, jolly nose, and laced cocked hat.

It is a common adage "as the old cock crows, the young one clucks." Born of the Whigs the Poor-Law Commissioners display the defects which have ruined the Whig Ministry, if not the Whig party. Whatever may have been the merits of that ministry, their warmest friends must admit their want of discretion, capriciously harsh carrying out of abstract principles, and strange combination of obstinacy and trick. These traits have characterised to a remarkable degree the Poor-Law Commissioners. Their indiscretion has been shewn in irritating the poor and the profession too by their arrangement of the districts and the Tender System—their capricious carrying out of abstract principles in their contradictory methods of enforcing their crotchets in different parts of the country, agreeing notwithstanding in one thing, being always arbitrary, insulting, and oppressive—their mingled obstinacy and trick, in pushing their arrangements under various disguises, and appealing to the results of fraud or intimidation, as conclusive evidence of success and satisfaction.

The "proof of the pudding is in the eating," and a very pretty pudding the Commissioners made of it. The poor hated their name—the medical profession were at daggers drawn with them—and a committee of the Commons House of Parliament quietly but explicitly condemned their system.

Now what did the Commissioners under these circumstances? Did they acknowledge their error, did they retrace their steps, did they conciliate opinion, did they bow to the decision of a parliamentary committee? Not at all. They took to their old course of shuffling and equivocating, and produced a Report intended to set them in their proper light of benefactors to the poor and friends to the profession.

So the commissioners being anxious to obtain the truth, and nothing but the truth of course, applied for it—to whom? To the paupers and the medical men? or to the aggrieved parties? Just the reverse. They applied to their own instruments, to the very people who were implicated. They asked them if there was dissatisfaction, if there was wrong, if things were not as they should be. Just as if we were to send a penny post letter to the gaoler to ask if the prisoner was treated kindly. We may guess the gaoler's answer, and we may guess the answer of the assistant-commissioners and guardians. Really Messieurs Commissioners you must think the world made up of excessive ninnies, to swallow anything so gross as this.

And yet even the assistant commissioners could not wholly gloss over the discontent.

"Seven of the assistant-commissioners have furnished the substance of the replies made by 242 unions, which, being situated in different parts of England and Wales, may be supposed fairly to represent the whole number hitherto formed. Of these 242, dissatisfaction is reported in sixty, on account of the low rate of medical remuneration; in thirty-one, on account of the size of districts or distance of paupers from medical advice; and in thirty-three, on account of neglect or inattention on the part of the medical officer.

So large an amount of dissatisfaction being admitted by those most anxious to conceal it,—it is difficult to comprehend the statement of the central board,—professedly founded on these returns, that 'there is *but little* dissatisfaction prevailing in reference to existing medical arrangements.'" 83.

It is difficult not to admire the imperturbable impertinence of these commissioners. The medical profession come forward in every way and publickly express their discontent and grievances—the poor, pretty unequivocally, express the same—their own agents and creatures employed in a sham inquiry report open disatisfaction in one-fourth of the unions they report from, and in the face of all this, the commissioners very coolly publish a book, for the purpose of asserting that there is "*but little* dissatisfaction." What would these people have? Will nothing short of an émeute convince them? Must we meet with naked arms and brandished catlins, and swear revenge or death? We tell the commissioners in so many words, the profession detest their measures. Surely that is enough. But no, the commissioners insist that we are very fond of them, and that they know our interests and our feelings better than we do ourselves.

But after this report of little dissatisfaction, come the recommendations of the commissioners, their small reform bill.

The particulars in which the commissioners profess their readiness to amend the medical arrangements are the following.

1. That the system of tender should now be abandoned.
2. That annual medical contracts should cease, and the union surgeons be appointed, as chaplains, clerks, and other paid officers are, for an indefinite period.

3. That the salaries should be computed on an annual list of the *regular* paupers, and on the separate illnesses of the *casual* paupers.

On this point, however, the commissioners differ from the parliamentary committee, Dr. Kay, and the medical witnesses, in confining the advantages of the pauper list to those in the actual receipt of general relief: also, in comprising the paupers of an entire district of several parishes in one list; thus imposing a uniform rate on parishes at different distances from medical advice.

4. That the remuneration for the pauper list should amount on the average to 6s. or 6s. 6d. per case, " subject to be augmented if the district is extensive;" and that the payments for those not included in the pauper list should be on a somewhat higher scale; " but the commissioners are inclined to think that it will not be found necessary to exceed 10s. per case."

" Midwifery and surgical operations of a serious character to be paid for by a separate charge for each case."

What merit there may be in these recommendations, is to be placed against a respectable *per contra*.

Amongst other things, their report endeavours to set aside, or to weaken the force of, several important recommendations of the parliamentary committee and the medical profession with respect to the extent of districts.

The commissioners assert that no rule or scale of limitation could be generally enforced; and that the division of the union into districts must, as now, be left to the uncontrolled discretion of the guardians.

On this the committee of the association observe;—

" The distribution of parochial duties must not be abandoned to the caprice of the guardians, who require, as many of them have confessed, some rule to guide them, in making a judicious medical division of the union.

Your committee object also to the main principle laid down by the commissioners for regulating the extent of districts, which is, ' that they should be sufficiently large to engage an important portion of the time and attention to the medical officer; and to create those responsibilities, those personal and pecuniary interests in the continuance to hold the office, which stimulate the officer to the efficient performance of his duty.'

Now, in your committee's last report, the responsibilities and personal interests involved in sedulous attention to the poor of a small district are shewn to outweigh those connected with a large one.

If, by ' an important portion of time,' &c. the commissioners mean a portion so considerable as shall preclude the medical practitioner from devoting his principal attention to private patients, their system will inevitably fail. For, since the salaries of union surgeons necessarily fall far short of the ordinary rate of professional remuneration, and are utterly inadequate to their maintenance, they are compelled to make private practice their first object, whether they are established practitioners, or are seeking for more lucrative employment, by means of the introduction resulting from the union appointment.

The wisest course, then, for the administrators of the law would be, not vainly to oppose this obvious and natural tendency, but to frame their plans accordingly; and to entrust to each medical officer no more ill-requited duty than he can properly perform with justice to his family and his professional station.

Your committee are confident that the greater the facility afforded to the medical officer, by small and convenient districts, for the performance of his duty, the stronger will be his inducement to hold office." 98.

To this we cordially assent, and it is impossible for any one not to feel indignant at the pertinacity with which the commissioners cling to arrangements reprobated by all but themselves. They may depend on it that, be they as obstinate as they may, they will fall, as their betters have done, in the war against public opinion. Nor will all their sophistry and shuffling save them. The unfitness of these men to superintend medical arrangements must be gross, when they can contend that, with the miserable pittance allowed for his professional attendance, the medical practitioner's time should be fully taken up with the paupers! Were it so, that same medical practitioner would infallibly soon be a pauper himself. It is strange that the commissioners should take such pains to write themselves down asses.

And can it be believed, that the commissioners after (on paper) abolishing the tender system, have the effrontery to defend it! If it is right, it should not be given up—if wrong, it should not be justified. Perhaps the commissioners insinuate that it *is* right, but they merely yield to prejudice. Rare commissioners, who alone possess the gift of wisdom, and before whom, all other men are but an ignorant rabble. Such is *our* benighted state that we desiderate that general ignorance rather than the precious learning of these Daniels.

But there is one insolent passage of their Report to be noticed.

" 'We do not mean to cast any reflection upon any particular person or class of persons; but we only state what might naturally be expected of any large body of men, when we say that their judgments were likely to be biassed against a new system by which their profits were lessened, their power curtailed, or their habits broken, and that they were likely to condemn it on slight evidence, and to give a ready acceptance to *ex parte* statements unfavourable to it.'

It should, however, be remarked, to the honour of the legal profession, that although their profits were materially diminished by the reduction of poor-law litigation, consequent on the new act, they have never taken any prominent part against the measure, or used the great abilities and influence which they possess for the purpose of discrediting it.' " 101.

The calumny upon the medical profession is only exceeded by the gross puff upon the legal. And by a lawyer it is written—his modesty being surpassed by his candour, and his candour by his independence. The law more generous, more humane, more liberal than physic! *That* was a discovery reserved for the commissioners. It is not medical men who are constantly presenting their gratuitous services to the needy and afflicted; no, it is the lawyer, good Samaritan! who is the friend to the distressed, the unpaid advocate of the poor, the liberal benefactor of all. Hence it is, perhaps, that his character in these respects stands so high—that his name is ever and popularly associated with all that is benevolent and good and kind. Gentle Chadwick, and thou art the impersonation of the benignity of the profession. We doubt not that in these pinching times thou wilt gladly resign half thy salary, and, without remuneration, as thou beneficently wishest us to do, devote *all thy* time to the paupers whom thou lovest, as Isaac Walton loved the frog he was impaling.

The absurdity of this libel is too palpable to render it necessary to reply to it, and yet we cannot altogether pass the sophistry of it. For the new poor-law simply took work away from the lawyer, whilst it made the medical man *work at a loss*. Two very different things. The one could not with any face complain. The other could not, if a man, be silent, especially when the insult of upstart officials was superadded to the injury. Let lawyers be so treated, and we venture to say there is *vice* enough in that meek profession to kick.

To make the case complete, it is necessary to advert to the *present* state of medical relief in England and Wales.

In September, 1840, the commissioners stated, in reply to an inquiry from the British Medical Association, that "they have hitherto perceived little disposition on the part of the boards of guardians to adopt their recommendations." And yet they hesitate to convert these recommendations into positive regulations.

The same disinclination of the guardians to amend their system is evident from the last (7th) annual Report of the poor-law commissioners.

It appears that a circular was addressed to the guardians in March last, inviting their attention to the previous suggestions of the commissioners, and inquiring as to the efficiency of the medical arrangements.

The replies from 117 unions (contained in that Report, p. 9) shew that the guardians, as on a former occasion, are for the most part perfectly satisfied with their own proceedings, and, in many instances, express their decided objection to any alteration.

In 48 of the 117 unions the guardians state their opinion that the districts are "not too large;" in 8 or 9 only do they profess to be ready to re-consider this point; in 60 they afford no information respecting the medical districts.

In 15 of the 117 unions, the guardians confess that the practice of advertising for tenders is still continued; in 12 only have they discontinued it; in a few it has never been adopted; but the majority of these unions make no return as to the continuance of appointment by tender.

A considerable number object to the "payment per case and pauper list," but without assigning their reasons.

It is highly improbable that the commissioners have selected the replies from these 117 unions to the prejudice of their system; it may therefore be fairly concluded that the remaining four-fifths of the unions in England and Wales would afford, to say the least, an equally unfavourable picture of the present medical arrangements.

The absurdity, then, of leaving the future administration of medical relief to the "improving care" of the guardians, is no less palpable than the necessity for legislative interference.

The Committee continue to receive information of medical districts so extensive as to incapacitate the medical officers for the proper performance of their duties, and to deprive the distant paupers of prompt relief: also of the selection of medical officers by tender, and of the appointment of imperfectly qualified practitioners.

One of the most flagrant instances of the "tender system" lately occurred in the Greenwich union, where the disgraceful pecuniary competition, on the part of the numerous medical candidates, and the low intrigue on the part of the guardians, exhibit in its worst aspect the "moral degradation" attendant on the system.

Other instances, recently recorded in the medical journals and provincial newspapers, might, if necessary, be cited; but the reports of the commissioners are quite sufficient to prove the continuance of abuses.

The same unexceptionable authority may be appealed to for proof that no general increase has taken place in poor-law medical remuneration since 1837.

The total expenditure in medical relief for the year ending March 25, 1840, is stated at £171,781.

This would scarcely afford 2¼d. per head on the population of 1831, and somewhat less on the real population of the year; while Mr. Farr shewed that, in 1837, the ratio of medical salaries to the population of eight counties was 3¼d. per head.

So with regard to the general expenditure for the relief of the poor in England and Wales, amounting to £4,756,965., the proportionate cost of medical relief in the same year was only 3.34 per cent.; whereas, in 1837, the proportion was calculated by Mr. Farr at 3.6 per cent.

It remains for us to notice the remedial measures proposed to be laid before the Parliament. The plan is in some sort engrafted on the Bill of Mr. Sergeant Talfourd. The following are its main provisions.

Clause A provides for the appointment of a medical "director," to superintend the medical department of the poor-law administration, subject to the approval of the commissioners.

This the committee think essential. We believe there would be some advantages in it, but we think it unlikely that such an appointment will be assented to by either Government or Parliament.

Clause B relates to the extent and population of districts.

It provides that no district shall include a larger population than ten thousand persons.

That districts of greater area than eight thousand acres (*about twelve square miles*) shall not include a population of more than four thousand persons.

That districts of greater area than one thousand acres (*about one and a half square mile*) shall not include a population of more than six thousand persons.

That districts of area less than one thousand acres may contain a population not exceeding ten thousand persons.

Clause C relates to the pauper lists. A quarterly revision is proposed, and a division of the claimants of medical relief.

Clause D relates to remuneration, and provides that the amount of such remuneration shall depend upon the average of the several quarterly numbers of persons whose names shall be on such list during the said ensuing year or any quarter thereof; and such rate of remuneration shall not be less than three shillings, nor more than four shillings for each individual of such average number.

And the said amount of remuneration, so determined, shall in the case of every parish in which the medical officer appointed to attend it shall not reside, and from which his residence shall be distant one mile, be augmented by the addition of one-fourth part for every mile of distance between the residence of such medical officer and the nearest part of such parish, such distance to be computed by the course of the nearest public carriage-way.

And further, the said amount of remuneration, determined as aforesaid, shall in like manner be augmented by the addition of one-fourth part in the case of every parish, the area of which shall exceed two thousand acres.

The committee observe:—In determining the rate of payment per head, the annual number of cases of illness and accident occurring in a given pauper population was estimated at about 67 per cent.

The annual sum for each *pauper* would thus be two-thirds of the average cost of medical attendance for each *case*. Now this, as regards the "regular" paupers, had been calculated by the medical witnesses at 5s., exclusive of the items of area and distance; the rate per head would therefore be equivalent to 3s. 4d., or a sum between 3s. and 4s., according to local circumstances.

The mileage charge would, doubtless, induce the guardians to commit the poor of the respective parishes to the nearest duly-qualified practitioner, and would thus aid the operation of clause B, in diminishing the extent of districts.

Clause E provides for the medical relief of the casual poor. It is proposed that each order should incur a payment of not less than 6s. nor more than 8s.; that is, *one-fourth* higher than the estimate for the cases of permanent paupers. And that the increase for distance should be in the same proportion as in the pauper list; viz. one-fourth (from 1s. 6d. to 2s.) additional for each mile.

"A reduction in the payment per case is proposed for cities and towns containing more than 10,000 inhabitants—a minimum of 4s. and maximum of 5s. being considered applicable to the circumstances of such populous places.

This clause contains a provision for empowering the parochial clergy to grant orders for medical relief, in addition to the parties authorised to perform this duty at present." 108.

But these orders are merely *as a loan* until the next meeting of the board of guardians who may allow or disallow them.

Clause F refers to the remuneration for workhouses. It provides that the amount of such remuneration shall depend upon the average, of the said ensuing year, weekly numbers of inmates of such workhouses, and shall not be less than 4s. 6d. nor more than 6s. for each individual of such average number.

Clause G enacts, that if by virtue of any order to be hereafter made by the poor-law commissioners, the guardians of any union shall think proper to provide medicines and other necessaries appertaining to medical relief, for the uses of the pauper patients of their union, paying competent persons to prepare and dispense the same, and appointing medical officers for the sole purpose of attending and prescribing for the poor of the union who may be sick or suffering from bodily injury; the said guardians shall in such case deduct one-half from the remuneration hereinbefore directed to be paid to the medical officer, exclusive of and without the affecting the augmentation made as aforesaid, on account of the extent of any parish, or the distance of such parish from his residence.

Clause H relates to the payment for attendance on difficult or protracted labours. It was at first proposed that the sum should be one guinea. But the committee have felt it due to the general opinion of the profession to alter the amount to two guineas.

Clause I provides a proper remuneration for serious surgical operations.

Clause K enacts, that the medical officer of every district shall, on or before the 25th day of March in every year after the passing of this Act, transmit to the medical commissioner a district report, stating the number of persons who shall have received medical relief during the preceding year within his district, the expenses of such relief, and the proportions and manner in which such expenses have been or will be defrayed, the distance of his own place of abode from the most remote inhabited part of his district, and if he shall not reside therein, in addition to such particulars as aforesaid, the distance of his place of abode from the nearest inhabited part of such district, and all such other matters as the poor-law commissioners shall by their orders from time to require to be included in such district report.

And that the medical commissioner shall once in every year prepare a general report, comprising the substance of such district reports, and all proceedings of the poor-law commissioners relating to medical relief in such year, and cause such general report to be annexed to the annual report of the poor-law commissioners, in order that the same may be submitted therewith, to one of the principal Secretaries of State, and laid therewith before both Houses of Parliament.

A provision is added for the payment of one guinea to the medical officer for each annual district report. As it would be impossible to enforce in every union a regulation compelling either the medical officers to reside within their districts, or the guardians to allot each parish to the nearest medical practitioner, it was considered essential that, in these reports, the extent of districts and the number of non-resident medical officers, should be annually brought under the cognizance of the Government and of Parliament.

Clause L provides that it shall be unlawful for the guardians of any poor-law union to attempt, by advertisement or other public notification, or in any manner whatsoever, to obtain tenders or offers relating to the remuneration specified in this Act to be given for the performance of the duties of medical officers, or to be received under any contract made, or to be made, according to the provisions of an Act of Parliament passed in the session of the third and fourth years of the reign of Her Majesty Queen Victoria, entituled, "An Act to extend the Practice of Vaccination."

Clause M enacts that no person shall hereafter be eligible to receive the appointment of medical officer of any district, not being duly qualified to practise as a surgeon and physician, or as a surgeon and apothecary, nor until he shall have been in medical and surgical practice, as principal or assistant, for a period of two years, unless at the time of passing this Act he shall have been in actual practice as a surgeon or apothecary for a period of five years.

The committee observe, in reference to their Bill, that objection has been taken to its length, many provisions, and complexity. But they reply that—

"Strongly impressed with the advantage of producing a simpler measure, and fully aware of the difficulty attending any attempt to define the remuneration—preferring also a judicious system of administration to legislation on matters of detail—your committee could have wished that Mr. Serjeant Talfourd's original clauses had been cordially and promptly supported by the whole profession. Under such favorable auspices, there would have been a reasonable prospect of their ultimate enactment. It was the denial of this general and vigorous support which led to the construction of the present clauses; and, however undesirable their length, repeated trials have convinced your committee of the utter futility of all attempts to determine, by one or two brief propositions, the various particulars of parochial remuneration, *on the principles indicated in the Report of the Parliamentary Committee.*" 110.

The Reporters do not conclude without a strong protest against both the construction and the working of the Vaccination Act. After setting forth the manner in which it was carried through both Houses, in spite of the opposition of the profession, an opposition unaided by the medical corporations, they go on to remark:—

"The operation of the act fully justified the apprehensions and predictions of your committee.

The poor-law commissioners at once proceeded to limit the ordinary remuneration of the district vaccinators to *eighteen-pence* for each successful case.

Your committee are prepared to admit that, under the former poor-law, the payments for pauper vaccination did not on the average exceed the present inadequate amount. And it is certain that but few unions, under the new poor-law, afforded a higher payment than that now proposed by the commissioners. Vaccination was in fact generally included in the gross medical salary; and to this circumstance, as well as to the negligence of the boards of guardians, may be partly attributed the increase of small-pox within the last few years.

It should, however, be remembered, that, prior to the Vaccination Act, the parochial or union authorities contracted for PAUPERS only, and for such of the working classes as were considered too poor to pay for vaccination.

Now, the privilege of gratuitous vaccination is extended to all who choose to apply for it, without reference to their circumstances or station; and the amendment (so called), which has just received legislative sanction, has removed the pauperising tendency of the gratuity.

It cannot, therefore, be matter of surprise that many who had been accustomed to pay their usual medical attendants, for vaccination, sums varying from half-a crown to half-a-guinea, should avail themselves of the new act, and apply to the public vaccinators.

The reduction in medical remuneration, which the commissioners have thus effected, is severely felt, not only by the bulk of the profession, but by the district vaccinators themselves, in consequence of their being required to furnish complicated weekly schedules, a quarterly registration of cases, certificates, and copies of register, which demand more than double the time and attention necessary for registration on a simpler plan." 114.

But all this is so much of a piece with the conduct of the commissioners throughout, as not to excite surprise. The consequence of this pennywise economy will be that the Vaccination Act will end in smoke, as far as the suppression of small-pox is concerned. Men who believe they are ill-treated, will never be hearty in a cause.

